# *IL1B*, *IL4R*, *IL12RB1* and *TNF* gene polymorphisms are associated with *Plasmodium vivax* malaria in Brazil

**DOI:** 10.1186/1475-2875-11-409

**Published:** 2012-12-07

**Authors:** Vinicius A Sortica, Maristela G Cunha, Maria Deise O Ohnishi, Jose M Souza, Ândrea KC Ribeiro-dos-Santos, Ney PC Santos, Sídia M Callegari-Jacques, Sidney EB Santos, Mara H Hutz

**Affiliations:** 1 Departamento de Genética, Universidade Federal do Rio Grande do Sul, Porto Alegre, RS, Brazil; 2 Laboratório de Genética Humana e Médica, Universidade Federal do Pará, Belém, Pará, Brazil; 3 Laboratório de Microbiologia e Imunologia, Instituto de Ciências Biológicas, Universidade Federal do Pará, Belém, Pará, Brazil; 4 Programa de Ensaios Clínicos em Malária, Instituto Evandro Chagas, Secretaria de Vigilância em Saúde, Ministério da Saúde, Ananindeua, Pará, Brazil; 5 Departamento de Estatística, Universidade Federal do Rio Grande do Sul, Porto Alegre, RS, Brazil

**Keywords:** Malaria, *Plasmodium vivax*, Immune system polymorphisms, Brazilian amazon, *IL1B*, *IL4R*, *IL12RB1*, *TNF*

## Abstract

**Background:**

Malaria is among the most prevalent parasitic diseases worldwide. In Brazil, malaria is concentrated in the northern region, where *Plasmodium vivax* accounts for 85% disease incidence. The role of genetic factors in host immune system conferring resistance/susceptibility against *P*. *vivax* infections is still poorly understood.

**Methods:**

The present study investigates the influence of polymorphisms in 18 genes related to the immune system in patients with malaria caused by *P*. *vivax*. A total of 263 healthy individuals (control group) and 216 individuals infected by *P*. *vivax* (malaria group) were genotyped for 33 single nucleotide polymorphisms (SNPs) in *IL1B*, *IL2*, *IL4*, *IL4R*, *IL6*, *IL8*, *IL10*, *IL12A*, *IL12B*, *IL12RB1*, *SP110*, *TNF*, *TNFRSF1A*, *IFNG*, *IFNGR1*, *VDR*, *PTPN22* and *P2X7* genes. All subjects were genotyped with 48 ancestry informative insertion-deletion polymorphisms to determine the proportion of African, European and Amerindian ancestry. Only 13 SNPs in 10 genes with differences lower than 20% between cases and controls in a Poisson Regression model with age as covariate were further investigated with a structured population association test.

**Results:**

The *IL1B* gene -5839C > T and *IL4R* 1902A > G polymorphisms and *IL12RB1* -1094A/-641C and *TNF* -1031 T/-863A/-857 T/-308 G/-238 G haplotypes were associated with malaria susceptibility after population structure correction (p = 0.04, p = 0.02, p = 0.01 and p = 0.01, respectively).

**Conclusion:**

*Plasmodium vivax* malaria pathophysiology is still poorly understood. The present findings reinforce and increase our understanding about the role of the immune system in malaria susceptibility.

## Background

Malaria remains one of the most important parasitic infections in the world with almost 250 million new cases diagnosed annually [1]. It is caused by infection with one or more of five species of *Plasmodium* parasites. *Plasmodium vivax* is the second most common cause of malaria in the world after *Plasmodium falciparum*, with high incidence in Asia, Central and South America causing high morbidity to these populations [2-5]. Traditionally, Brazil has been responsible for almost half of all cases of malaria in Latin America. In 2010, 334,618 cases of malaria (283,384 caused by *P*. *vivax*) were reported in this country [1,6].

*Plasmodium vivax* has unique biological features that distinguish it as a species. The most obvious features that distinguish *P*. *vivax* from *P*. *falciparum* include the development of dormant forms (hypnozoites) in the liver that cause subsequent infections in the blood called relapses, which add a substantial number of cases to the general burden of the disease and present one of the most challenging bottlenecks for vivax malaria eradication [7].

A sensitive balance between pro- and anti-inflammatory immune response, primarily mediated by cytokines released by T helper (Th) cells and macrophages, is necessary for an adequate response to malaria. An early pro-inflammatory response mediated by a set of Th1 cells and macrophages helps to reduce parasitaemia; however, a second anti-inflammatory response mediated by a set of Th2 cells and monocytes is required to control malaria by preventing organ damage and more severe symptoms. Timing and intensity between Th1 and Th2 response and auto-regulation can influence both pathology of infection and its progress [8-10]. Influence of the immune system gene polymorphisms with resistance/susceptibility and severity in *P*. *falciparum* malaria in Africa and Asia have been reported [11], however the influence of these polymorphisms in *P*. *vivax* infection is still poorly investigated. In patients with *P*. *vivax* malaria from India, two single nucleotide polymorphisms (SNP) in the *TNF* promoter (−308 G > A and -1031C > T) were associated with tumour necrosis factor (TNF) levels and clinical symptoms but not with susceptibility [12]. A recent work, demonstrated an association of +874A > T in *IFNG* gene with Interferon gamma (IFN-γ) plasma levels but not with -1082 T > C in *IL10* gene with interleukin (IL) 10 plasma levels in patients infected by *P*. *vivax* from Brazil [13].

Brazil has a peculiar epidemiological situation, as one of the few countries around the world with *P*. *vivax* predominance [6]. Data on susceptibility to *P*. *vivax* infection allied to several particularities of the Brazilian Amazonian region, including the diverse genetic background of its population, indicate that the generalization of findings from Southeast Asia may not be appropriate for this region [6]. The present work aims to investigate the influence of 33 SNPs in genes related to the immune system and susceptibility to *P*. *vivax* malaria in an Amazonian population.

## Methods

### Study population

A total of 216 patients were diagnosed with *P*. *vivax* malaria at the Evandro Chagas Institute, Belém, Pará between 2002–2009. All patients were diagnosed by thick blood smear as recommended by the Brazilian Ministry of Health [14]. The patients received the standard treatment 1,500 mg of chloroquine associated with 210 mg of primaquine in seven days. Between 2006 and 2008, 263 healthy controls were recruited at the Federal University of Pará. Unrelated subjects were interviewed at the Human and Medical Genetics Laboratory and agreed to donate DNA samples to compose a population sample for genetic studies. The individuals enrolled in the study were born in Pará state in the Brazilian Amazonian region. Patients and controls provided their written informed consent to participate in this study. The Ethics Committees of the Evandro Chagas Institute and Federal University of Pará approved the study protocol.

### Genotyping

Genomic DNA was extracted from peripheral blood leukocyte subjects using proteinase K digestion and standard phenol-chloroform procedures [15]. All subjects were genotyped for a set of 48 bi-allelic short insertion/deletion (indels) polymorphisms, validated as ancestry informative markers. The genotyping procedure was performed by three multiplex reactions as described elsewhere [16].

The 33 immune system gene SNPs were determined by allelic discrimination with Taqman 5’-nuclease assays. A total of 30 polymorphisms were genotyped with validated genotyping assays (Real Time PCR, Applied Biosystems, CA, USA). Three variants -174C > G (rs1800795), -863A > C (rs180030) and 874A > T (rs2430561) were genotyped by custom genotyping assay by design (Applied Biosystems, CA, USA). All assays were genotyped according to the manufacturer’s recommended protocol.

### Statistical analyses

Allele and genotype frequencies were estimated by gene counting. Deviation from Hardy-Weinberg equilibrium was assessed by Qui-square tests with Bonferroni correction. Haplotype frequencies and linkage disequilibrium were estimated with PHASE 2.1.1 [17]. Differences between malaria and control samples on age and ancestry were estimated by Mann–Whitney tests. The individual proportions of European, African and Amerindian genetic ancestry were estimated using the STRUCTURE software 2.3.3 assuming three parental populations (Europeans, Africans and Amerindians), and running with 200,000 burn-in period and 200,000 Markov Chain Monte Carlo repetitions after burning [18]. Poisson regressions were performed to assess the association between polymorphisms or haplotypes and age. The association between malaria cases and controls was performed using the STRAT software with 10,000 simulations [19]. STRAT utilizes the STRUCTURE output to test for association in the presence of population stratification based on individual ancestry information. Mann–Whitney, Qui-square and Poisson Regression tests were performed using the SPSS18.0 statistical package for Windows®. Statistical significance was defined as a two-tailed P-value < 0.05.

## Results

The 33 SNPs investigated, their location in the gene and the allele frequencies observed in malaria cases and controls are shown in Additional file 1. The genotype distribution did not deviate significantly from Hardy-Weinberg equilibrium in both samples (Additional file 2).

Age and mean ancestry proportions of the subjects enrolled in the study are shown in Table 1. The control group was younger (32.8 ± 16.5 years) and presented a larger proportion of European ancestry (0.436 ± 0.13) than the malaria patients (35.4 ± 15.3 years; 0.412 ± 0.11 respectively). These differences were statistically significant between groups (p = 0.003 and p = 0.037, respectively). Due to this significant difference in age, a Poisson regression analysis using age as a covariate was performed. Only the polymorphisms with P <0.20 between malaria and control samples were chosen for further analyses (Additional file 3).

**Table 1:** Age and genetic ancestry of malaria and control subjects

**Characteristics**	**Controls**	**Malaria**	**P value**^**a**^
N	263	216	
Age (y)	32.8 ± 16.5	35.4 ± 15.3	0.003
Genetic Ancestry			
African	0.245 ± 0.10	0.246 ± 0.09	0.6
European	0.436 ± 0.13	0.412 ± 0.11	0.03
Native American	0.319 ± 0.11	0.342 ± 0.12	0.06

Values for age and genetic ancestry are expressed as mean ± SD.

^a^ Mann–Whitney test p values.

Because ancestry proportions differ between malaria patients and controls (Table 1), the association between 13 SNPs, chosen from the Poisson regression analyses, was performed using the STRAT software (Table 2). After population structure correction, only *IL1B* -5839C > T and *IL4R* 1902A > G polymorphisms were associated with malaria susceptibility. The *IL1B* -5839C and *IL4R* 1902A alleles are 8.2% and 6.2% respectively more frequent in malaria patients than in controls. Haplotype association tests adjusted for population stratification by the STRAT software are shown in Table 3. *IL12RB1* and *TNF* haplotypes were associated with malaria susceptibility. The *IL12RB1*AC (−1094/-641) haplotype is only present in the malaria sample whereas the *TNF* TATGG (−1031/-863/-857/-308/-238) haplotype is 2.5% more frequent in individuals with malaria.

**Table 2:** Structured population association test between malaria and control samples

**Gene**	**SNP**	**dbSNP ID**	**Allele**	**Frequency (%)**		**P**_**Qui**_^**2**^	**P**_**Strat**_
				**Control**	**Malaria**		
*IL1B*	−5839C > T	rs1143629	C	46.2	54.4	0.01	**0.04**
*IL4R*	1902A > G	rs1801275	A	32.5	38.7	0.04	**0.02**
*IL6*	−174C > G	rs1800795	C	20.9	17.8	0.25	0.50
*IL10*	−592A > C	rs1800872	A	34.2	37.7	0.27	0.40
*IL12B*	458A > G	rs2546890	A	40.1	33.6	0.04	0.12
*IL12RB1*	−1094A > G	rs375947	G	23.4	20.9	0.36	0.19
	−641C > T	rs11575934	G	20.8	19.9	0.72	0.79
SP110	14622C > T	rs2114592	T	8.2	9.3	0.55	0.75
*TNF*	−1031C > T	rs1799964	C	24.0	21.9	0.44	0.10
	−238A > G	rs361525	A	7.4	6.1	0.44	0.37
	−857C > T	rs1799724	T	14.4	15.0	0.79	0.12
IFNG	874A > T	rs2430561	A	25.8	28.3	0.38	0.29
*IFNGR1*	−56 T > C	rs2234711	C	39.8	38.1	0.59	0.71

**Table 3:** Structured population association test for haplotypes association between malaria and control samples

**Gene**	**Haplotype**	**Allele**	**Frequency (%)**		**P**_**Qui**_^**2**^	**P**_**Strat**_
			**Control**	**Malaria**		
*IL1B*	−5839/-31/-511	TTG	49.0	42.8	0.02	0.07
		TTA	0.2	0.2		
		TCG	2.9	0.9		
		TCA	1.5	1.6		
		CTG	0.2	1.5		
		CCA	46.2	53.0		
*IL10*	−592A/-819/-1082	CCA	36.3	39.3	0.17	0.57
		CCG	29.2	22.8		
		CTA	0.4	0.2		
		ATA	34.1	37.5		
		ATG	0	0.2		
*IL12B*	159/458/735	CGC	31.8	36.3	0.09	0.22
		CGT	1.4	4.0		
		CAC	0.7	0.2		
		CAT	2.7	2.4		
		AGC	11.6	10.1		
		AGT	14.1	15.8		
		AAC	0.2	0.5		
		AAT	37.5	30.7		
*IL12RB1*	−1094/-641	AT	76.7	77.8	0.04	**0.01**
		AC	0	1.4		
		GT	2.5	2.3		
		GC	20.8	18.5		
*TNF*	−1031/-863/-857/-308/-238	TCCGG	57.8	61.4		
		TCCGA	1.6	0.2		
		TCCAG	6.6	6.7		
		TCTGG	11.1	9.8		
		TCTGA	0.5	0.2		
		TCTAG	0	0.2		
		TACGG	1.3	0.2	0.07	**0.01**
		TATGG	1.3	3.8		
		CCCGG	1.6	0.2		
		CCCGA	0.5	1.0		
		CCTGG	0.8	0.2		
		CACAA	16.3	15.7		
		CACGA	0.3	0.2		
		CATGG	0.3	0.2		
*IFNGR1*	−56 /611	TA	32.7	36.1	0.28	0.57
		TG	28.1	25.9		
		CA	38.0	37.7		
		CG	1.2	0.3		

## Discussion

In a gene-based association study with 18 candidate genes for malaria susceptibility using 33 SNPs as genetic markers, this study demonstrated that *IL1B*, *IL4R*, *IL12RB1* and *TNF* genes were associated with susceptibility to *P*. *vivax* malaria in a population of Pará state, Brazil.

Cytokines are immunomodulatory proteins produced by a wide variety of cells, and with very complex activities. A functional cytokine network is a central element in the homeostasis of the immune response and its alteration may lead to an abnormal immune response. Hence, recent interest has focused upon genes regulating the cytokine expression; in particular on gene polymorphisms that may influence the levels of expression and therefore the overall immune response. Despite evidence demonstrating the importance of *IL1B*, *IL4R*, *IL12RB1* and *TNF* genes in *P*. *falciparum* malaria pathology, the influence of these variants in *P*. *vivax* infections is unknown.

Independent studies reported differences in immune system gene polymorphisms frequencies in distinct malaria-endemic regions [20]. The relevance of these polymorphisms in malaria infections could differ between distinct genetic background populations or etiologic agents, highlighting the importance of studies in different endemic regions. Due to the high admixed nature of the Brazilian population and its substructuring consequences in genetic association studies, this study dealt with this issue with extreme care. The population from Pará state has European, African and Amerindian ancestral groups [16,21], so that a structured population association test to avoid genetic bias in the analysis was employed to provide reliable results for this specific population.

The IL-1β, IL-12 and the TNF together with IFN-γ are the major cytokines in pro-inflammatory Th1 immune response. IL-1β is predominantly secreted by monocytes and macrophages in initial immune response against infections [22-24] and helps to modulate the expression of IFN-γ and promote the polarization to Th17 immune response in certain circumstances [25,26]. IL-12 promotes IFN-γ production by T and natural killer (NK) cells and exerts its biological function through binding to the heteromeric interleukin 12 receptor (IL-12R) β1 and β2. The deficiency in IL-12R expression interferes in IL-12 functions and is associated with severe infection in humans [27,28]. TNF is produced by monocytes and macrophages and its role in malaria pathology was investigated due to reports of high levels of this cytokine in cerebral malaria patients [29].

*IL1B* gene was associated with *P*. *falciparum* malaria in African populations only [30,31]. The present study is the first to report the association of -5839C > T SNP promoter with *P*. *vivax* malaria susceptibility. The -5839C allele presented a higher frequency in malaria patients then in controls. Despite that, the function of this intronic SNP is not completely elucidated, variability of this important pro-inflammatory gene could represent an important factor in immune regulation. The association of haplotype -31C/-511A in *IL1B* gene promoter with severe malarial anaemia and circulating IL-1β low levels in children with *P*. *falciparum* malaria from Kenya have been shown recently [32], however, the *IL1B* -31C > T polymorphism was not associated with cerebral malaria in Thailand [33]. In the present study these two SNPs were not associated with vivax malaria, demonstrating a possible difference in the contribution of these polymorphisms to malaria pathology among populations and parasites. Complementary studies in *IL1B* gene and IL-1β levels are important to help understand how this gene influences malaria susceptibility and severity.

Recent work in Kenyan patients infected with *P*. *falciparum* demonstrated that *IL12RB1* rs4229774 and rs383483 polymorphisms were associated with protection against severe malarial anaemia and high parasitaemia levels, but not susceptibility [34]. The present study reports the association of *IL12RB1* -1094A/-641C haplotype with *P*. *vivax* malaria susceptibility. Despite the lower frequency in the study subjects, this haplotype is present only in the individuals with malaria, suggesting a possible influence in malaria response. The -641C allele leads to a missense variant (i.e. encodes a different amino acid) and can modify the receptor properties and interfere with IL-12 ligation and function. These results suggest that *IL12RB1* variants are important in malaria susceptibility and severity. Further studies will be necessary to better understand the *IL12RB1* influence on susceptibility and severity in *P*. *vivax* malaria.

Polymorphisms in *TNF* gene promoter have been reported to be associated with symptoms and severity of *P*. *falciparum* malaria in different African and Asian populations [35-39]. The present work demonstrated that the TATGG (−1031/-863/-857/-308/-238) *TNF* haplotype is associated with *P*. *vivax* malaria in a Brazilian population. TNF is an important pro-inflammatory cytokine and the TATGG haplotype diverge in two alleles (−1031C and -308A) associated with TNF levels in vivax malaria infection in India [12]. In that work it was hypothesized that -1031C and -308A alleles are rare in Indian malaria patients due to a possible protective effect. The presence of -1031 T and -308 G alleles in TATGG haplotype could be an important factor in vivax malaria susceptibility.

Interleukin 4 receptor (IL-4R), together with IL-4 and IL-13 are important Th2 anti-inflammatory immune response modulators. IL-4R is the principal receptor of these interleukins, and when it is blocked IL-4 and IL-13 function is aborted preventing Th2 immune response modulation [40]. Only a few works investigated IL-4R variants influence on malaria infections. In the present investigation an association of 1902A > G SNP with malaria susceptibility was observed. The 1902 G allele create a missense variant and potentially can modify the receptor properties and interfere with IL-4 and IL-13 functions. It has been shown that the immune response via IL-4, IL4R and IL-13 pathway is important to prevent malaria infection in mice [41,42]. These studies demonstrated that knockout mice for *IL4* and *IL4R* genes have high resistance to malaria liver stage caused by sporozoites. The absence of modulation mediated by IL-4 to Th2 immune response, the Th1 response mediated by IFN-γ is maintained and promotes a rapid cellular response against sporozoites. The present study shows that 1902A allele is more frequent in malaria patients, although 1902 G presents a higher overall frequency in the study population, 1902A allele could influence the co-regulation between Th1 and Th2 immune response against malaria infections. New complementary studies will be necessary to elucidate the *IL4R* gene importance in vivax malaria infection.

*IL4*, *IL10*, *IL12B*, *IFNG* and *IFNGR1* genes polymorphisms were associated with symptoms and severity of *P*. *falciparum* malaria in studies from African and Asian populations [11]. In the present study, polymorphisms in these genes were not associated with susceptibility to malaria caused by *P*. *vivax*. Differences in malaria pathophysiology caused by these two species of parasites could be a possible explanation for the divergences reported. *Plasmodium falciparum* malaria presents a more acute form of infection reaching more than 50% of erythrocytes leading to cyto-adherence, organ damaged, severe malarial anaemia and cerebral malaria. Malaria caused by *P*. *vivax* is characterized by a long incubation period and milder initial symptoms. The parasite infects approximately 2 to 5% of erythrocytes, and can remain dormant in the liver as hypnozoites leading to subsequently relapses [43]. Studies have linked high levels of pro-inflammatory cytokines, such as TNF, IL-1β, IL-6 and IFN-γ in *P*. *falciparum* infections [44-47] and TNF, IL-1β, IL-2, IL-4, IL-6, IL-8, IL-10 e IL-12 cytokines with *P*. *vivax* infections [13,48-50]. These differences in cytokine profile and gene polymorphisms should reflect a distinct dynamic between the regulatory pathway of pro- and anti-inflammatory cytokines in *P*. *falciparum* and *P*. *vivax* malaria response, pathology and outcome.

## Conclusions

Knowledge of the relationships between genetics, susceptibility to and severity of malaria infections is essential to identify subjects at higher risk and to develop specific preventive measures. This study showed that genetic polymorphisms of some interleukins involved in the immune response to *P*. *vivax* infection influence susceptibility to malaria. The present findings reinforce and increase our understanding about the role of the immune system in malaria susceptibility. However, to expand knowledge about the actions of the immune system in the pathophysiology of vivax malaria it is necessary to conduct population-based longitudinal studies.

## Abbreviations

Th: T helper; SNP: Single nucleotide polymorphism; TNF: Tumour necrosis factor; IFN-γ: Interferon gamma; IL: Interleukin; Indels: Insertion/deletion; NK: Natural killer; IL-12R: Interleukin 12 receptor; IL-4R: Interleukin 4 receptor.

## Competing interests

The authors declare that they have no competing interests.

## Authors’ contributions

VAS designed the research project, carried out the laboratory assays, and wrote the manuscript; MGC participated in the study design, planning and data collection, revised the manuscript for important intellectual content and approved the version to be published; MDOO participated in the study design, planning and data collection, performed the malaria diagnosis and revised the manuscript for important intellectual content and approved the version to be published; JMS participated in the study design, planning and data collection, performed the malaria diagnosis and revised the manuscript for important intellectual content and approved the version to be published; NPCS participated in the study design, planning and data collection, performed the malaria diagnosis and revised the manuscript for important intellectual content and approved the version to be published; AKCR participated in the study design, planning and data collection, revised the manuscript for important intellectual content and approved the version to be published; SMC-J participated in the study design, planning and performed the statistical analysis and approved the version to be published; SEBS genotyped the ancestry informative markers, revised the manuscript for important intellectual content, and approved the version to be published; MHH conceived the study, and participated in its design and coordination, analysed the data, and wrote the final version of the article. All authors read and approved the final manuscript.

## Supplementary Material

Additional file 1 **Table S1. **List of SNPs investigated in the present study. List of SNPs investigated, their location in the gene, pubmed database SNP identification, manufacturer’s assay identification and allele frequencies in case and control groups. Click here for file

Additional file 2 **Table S2. **Control and malaria group genotypic frequencies. Genotypic frequencies of all SNPs in case and control groups. Click here for file

Additional file 3 **Table S3. **Poisson Regression for association between malaria and control samples controlling for age. Poisson Regression results for association between malaria and control samples controlling for age. Click here for file
