# Supplementary material for: IL1B, IL4R, IL12RB1 and TNF gene polymorphisms are associated with Plasmodium vivax malaria in Brazil
Source: Malar J. 2012 Dec 7;11:409. doi: 10.1186/1475-2875-11-409 (PMC3537609; doi:10.1186/1475-2875-11-409)
Supplement: Additional file 1 — Table S1. List of SNPs investigated in the present study. List of SNPs investigated, their location in the gene, pubmed database SNP identification, manufacturer’s assay identification and allele frequencies in case and control groups. [file 1475-2875-11-409-S1.pdf]

Additional Table 1. List of SNPs investigated in the present study

Gene	Location	SNP	dbSNP ID	Assay ID	Allele	Frequency	
						Control	Malaria
IL1B	intron	-5839C>T	rs1143629	C___1839945_1_	T	0.538	0.456
					C	0.456	0.544
	5' upstream	-31C>T	rs1143627	C___1839944_10	C	0.504	0.496
					T	0.556	0.444
	5' upstream	-511A>G	rs16944	C___1839943_10	G	0.521	0.451
					A	0.479	0.549
IL2	5' upstream	-330G>T	rs2069762	C__15859930_10	T	0.712	0.641
					G	0.288	0.359
IL4	5' upstream	-590C>T	rs2243250	C__16176216_10	C	0.587	0.530
					T	0.413	0.470
IL4R	missense	1902A>G	rs1801275	C___2351160_20	G	0.675	0.613
					A	0.325	0.387
IL6	5' upstream	-174C>G	rs1800795*		G	0.791	0.822
					C	0.209	0.178
IL8	5' upstream	-251A>T	rs4073	C__11748116_10	A	0.456	0.458
					T	0.544	0.542
IL10	5' upstream	-592A>C	rs1800872	C___1747363_10	A	0.342	0.377
					C	0.658	0.623
	5' upstream	-1082T>C	rs1800896	C___1747360_10	G	0.707	0.771
					A	0.293	0.229
	5' upstream	-819C>T	rs1800871	C___1747362_10	T	0.345	0.379
					C	0.655	0.621
IL12A	3' UTR	121G>A	rs568408	C___2423981_10	A	0.108	0.089
					G	0.892	0.911
IL12B	5' upstream	735T>C	rs7709212	C__31985592_10	T	0.440	0.469
					C	0.560	0.531
	intron	458A>G	rs2546890	C__15894458_10	A	0.401	0.336

					G	0.599	0.664
	3' UTR	159A>C	rs3212227	C___2084293_10	C	0.385	0.430
					A	0.615	0.570
IL12RB1	missense	-1094A>G	rs375947	C____795442_1_	A	0.766	0.791
					G	0.234	0.209
	missense	-641C>T	rs11575934	C__27529556_10	C	0.208	0.199
					T	0.792	0.801
SP110	intron	14622C>T	rs2114592	C__15816049_10	C	0.918	0.907
					T	0.082	0.093
	missense	1274C>T	rs3948464	C___2915061_10	C	0.880	0.863
					T	0.120	0.137
TNF	5' upstream	-308C>T	rs1800629	C___7514879_10	A	0.076	0.073
					G	0.924	0.927
	5' upstream	-1031C>T	rs1799964	C___7514871_10	C	0.240	0.219
					T	0.760	0.781
	5' upstream	-238A>G	rs361525	C___2215707_10	A	0.074	0.061
					G	0.926	0.939
	5' upstream	-863A>C	rs1800630*		A	0.210	0.202
					C	0.790	0.798
	5' upstream	-857C>T	rs1799724	C__11918223_10	T	0.144	0.150
					C	0.856	0.850
TNFRSF1A	intron	303A>G	rs4149622	C__32131594_10	A	0.857	0.832
					G	0.143	0.168
IFNG	intron	874A>T	rs2430561*		T	0.742	0.717
					A	0.258	0.283
IFNGR1	5' upstream	611C>T	rs1327474	C___2523634_10	G	0.292	0.261
					A	0.708	0.739
	5' UTR	-56T>C	rs2234711	C__11693991_10	T	0.602	0.619
					C	0.398	0.381
VDR	missense	FokI	rs10735810	C__12060045_20	C	0.651	0.645

					T	0.349	0.355
	synonymous	TaqI	rs731236	C___2404008_10	T	0.729	0.701
					C	0.271	0.299
	intron	BsmI	rs1544410	C___8716062_10	G	0.719	0.726
					A	0.281	0.274
PTPN22	missense	R630W	rs2476601	C__16021387_20	G	0.949	0.951
					A	0.051	0.049
P2X7	missense	1513T>G	rs3751143	C__27495274_10	T	0.797	0.844
					G	0.203	0.156

*Custom assay
