# Supplementary material for: IL1B, IL4R, IL12RB1 and TNF gene polymorphisms are associated with Plasmodium vivax malaria in Brazil
Source: Malar J. 2012 Dec 7;11:409. doi: 10.1186/1475-2875-11-409 (PMC3537609; doi:10.1186/1475-2875-11-409)
Supplement: Additional file 2 — Table S2. Control and malaria group genotypic frequencies. Genotypic frequencies of all SNPs in case and control groups. [file 1475-2875-11-409-S2.pdf]

Additional Table 2. Control and malaria group genotypic frequencies

Gene	dbSNP ID	N	Genotypes		
IL1B					
-5839C>T	rs1143629		TT	TC	CC
Control		263	83 (31.5)	117 (44.5)	63 (24.0)
Malaria		216	48 (22.2)	101 (46.8)	67 (31.0)
-31C>T	rs1143627		CC	CT	TT
Control		263	80 (30.4)	105 (39.9)	78 (29.7)
Malaria		215	72 (33.5)	95 (44.2)	48 (22.3)
-511A>G	rs16944		GG	GA	AA
Control		261	80 (30.7)	112 (42.9)	69 (26.4)
Malaria		214	50 (23.4)	93 (43.5)	71 (33.1)
IL2					
-330G>T	rs2069762		TT	GT	GG
Control		262	130 (49.6)	113 (43.1)	19 (7.3)
Malaria		216	84 (38.9)	109 (50.5)	23 (10.6)
IL4					
-590C>T	rs2243250		CC	CT	TT
Control		263	88 (33.5)	133 (50.5)	42 (16.0)
Malaria		216	58 (26.9)	113 (52.3)	45 (20.8)
IL4R					
1902A>G	rs1801275		GG	GA	AA
Control		263	131 (49.8)	93 (35.4)	39 (14.8)
Malaria		216	78 (36.1)	109 (50.5)	29 (13.4)
IL6					
-174C>G	rs1800795		GG	GC	CC
Control		263	162 (61.6)	92 (35.0)	9 (3.4)
Malaria		216	147 (68.1)	61 (28.2)	8 (3.7)
IL8					
-251A>T	rs4073		AA	AT	TT
Control		263	56 (21.3)	128 (48.7)	79 (30.0)
Malaria		212	43 (20.3)	108 (50.9)	61 (28.8)
IL10					
-592A>C	rs1800872		AA	AC	CC
Control		263	35 (13.3)	110 (41.8)	118 (44.9)
Malaria		216	28 (13.0)	107 (49.5)	81 (37.5)
-1082T>C	rs1800896		GG	GA	AA
Control		263	133 (50.6)	106 (40.3)	24 (9.1)
Malaria		216	128 (59.3)	77 (35.6)	11 (5.1)
-819C>T	rs1800871		TT	TC	CC
Control		262	36 (13.7)	109 (41.6)	117 (44.7)
Malaria		215	29 (13.5)	105 (48.8)	81 (37.7)
IL12A					
121G>A	rs568408		AA	AG	GG
Control		223	5 (2.2)	38 (17.0)	180 (80.8)
Malaria		213	3 (1.4)	32 (15.0)	178 (83.6)
IL12B					
735T>C	rs7709212		TT	TC	CC
Control		226	70 (31.0)	113 (50.0)	43 (19.0)
Malaria		213	66 (31.0)	94 (44.1)	53 (24.9)
458A>G	rs2546890		AA	AG	GG
Control		237	49 (20.7)	92 (38.8)	96 (40.5)
Malaria		214	28 (13.1)	88 (41.1)	98 (45.8)
159A>C	rs3212227		AA	CA	AA
Control		260	49 (18.8)	102 (39.2)	109 (42.0)
Malaria		213	42 (19.7)	99 (46.5)	72 (33.8)
IL12RB1					

-1094A>G	rs375947		AA	AG	GG
Control		263	160 (60.8)	83 (31.6)	20 (7.6)
Malaria		215	132 (61.4)	76 (35.3)	7 (3.3)
-641C>T	rs11575934		GG	GA	AA
Control		262	17 (6.5)	75 (28.6)	170 (64.9)
Malaria		214	6 (2.8)	73 (34.1)	135 (63.1)
SP110					
14622C>T	rs2114592		CC	CT	TT
Control		262	221 (84.4)	39 (14.8)	2 (0.8)
Malaria		215	176 (81.9)	38 (17.6)	1 (0.5)
1274C>T	rs3948464		CC	CT	TT
Control		263	201 (76.4)	61 (23.2)	1 (0.4)
Malaria		215	160 (74.1)	53 (24.5)	3 (1.4)
TNF					
-308C>T	rs1800629		AA	AG	GG
Control		263	2 (0.8)	36 (13.7)	225 (85.5)
Malaria		213	1 (0.5)	29 (13.6)	183 (85.9)
-1031C>T	rs1799964		CC	CT	TT
Control		263	12 (4.6)	102 (38.8)	149 (56.6)
Malaria		215	9 (4.2)	76 (35.3)	130 (60.5)
-238A>G	rs361525		AA	AG	GG
Control		224	3 (1.3)	27 (12.1)	194 (86.6)
Malaria		214	3 (1.4)	20 (9.3)	191 (89.3)
-863A>C	rs1800630		AA	AC	CC
Control		252	22 (8.7)	62 (24.6)	168 (66.7)
Malaria		213	21 (9.9)	44 (20.7)	148 (69.5)
-857C>T	rs1799724		TT	TC	CC
Control		205	8 (3.9)	43 (21.0)	154 (75.1)
Malaria		213	6 (2.8)	52 (24.4)	155 (72.8)
TNFRSF1A					
303A>G	rs4149622		AA	AG	GG
Control		263	193 (73.4)	65 (24.7)	5 (1.9)
Malaria		214	146 (68.2)	64 (29.9)	4 (1.9)
IFNG					
874A>T	rs2430561		TT	TA	AA
Control		262	144 (55.0)	101 (38.5)	17 (6.5)
Malaria		214	111 (51.9)	85 (39.7)	18 (8.4)
IFNGR1					
-611(C>T)	rs1327474		GG	GA	AA
Control		209	21 (10.0)	80 (38.3)	108 (51.7)
Malaria		213	15 (7.0)	81 (38.1)	117 (54.9)
-56T>C	rs2234711		TT	TC	CC
Control		250	99 (39.6)	103 (41.2)	48 (19.2)
Malaria		214	82 (38.3)	101 (47.2)	31 (14.5)
VDR					
FokI	rs10735810		CC	CT	TT
Control		259	116 (44.8)	105 (40.5)	38 (14.7)
Malaria		214	90 (42.1)	96 (44.8)	28 (13.1)
TaqI	rs731236		TT	TC	CC
Control		262	146 (55.7)	90 (34.4)	26 (9.9)
Malaria		179	84 (46.9)	83 (46.4)	12 (6.7)
BsmI	rs1544410		GG	GA	AA
Control		263	139 (52.9)	100 (38.0)	24 (9.1)
Malaria		210	107 (51.0)	91 (43.3)	12 (5.7)
PTPN22					
R630W	rs2476601		GG	GA	AA
Control		263	236 (89.7)	27 (10.3)	0
Malaria		215	21 (9.8)	194 (90.2)	0

P2X7					
1513T>G	rs3751143		TT	TG	GG
Control		263	164 (62.4)	91 (34.6)	8 (3.0)
Malaria		211	148 (70.1)	60 (28.5)	3 (1.4)
