# Supplementary material for: IL1B, IL4R, IL12RB1 and TNF gene polymorphisms are associated with Plasmodium vivax malaria in Brazil
Source: Malar J. 2012 Dec 7;11:409. doi: 10.1186/1475-2875-11-409 (PMC3537609; doi:10.1186/1475-2875-11-409)
Supplement: Additional file 3 — Table S3. Poisson Regression for association between malaria and control samples controlling for age. Poisson Regression results for association between malaria and control samples controlling for age. [file 1475-2875-11-409-S3.pdf]

Additional Table 3. Poisson Regression for association between malaria and control samples controlling for age

Gene	SNP	dbSNP ID	Risk Genotype	P value
IL1B	-5839C>T	rs1143629	TC	**0.02**
			CC	**0.08**
	-31C>T	rs1143627	CT	0.25
			TT	0.41
	-511A>G	rs16944	GA	0.80
			AA	0.75
IL2	-330G>T	rs2069762	GT	0.26
			TT	0.30
IL4	-590C>T	rs2243250	CT	0.95
			TT	0.87
IL4R	1902A>G	rs1801275	AG	**0.009**
			GG	0.31
IL6	-174C>G	rs1800795	GC	**0.01**
			CC	0.34
IL8	-251A>T	rs4073	AT	0.72
			TT	0.59
IL10	-592A>C	rs1800872	CA	0.42
			CC	**0.15**
	-1082T>C	rs1800896	GA	0.32
			GG	0.61
	-819C>T	rs1800871	CT	0.25
			TT	0.73
IL12A	121G>A	rs568408	AG	0.93
			GG	0.98
IL12B	735T>C	rs7709212	TC	0.30
			TT	0.31
	458A>G	rs2546890	AG	**0.01**
			GG	**0.02**
	159A>C	rs3212227	AC	0.22
			AA	0.67
IL12RB1	-1094A>G	rs375947	GA	**0.009**
			GG	**0.003**
	-641C>T	rs11575934	GA	0.56
			AA	**0.07**
SP110	14622C>T	rs2114592	CT	**0.13**
			CC	**0.15**
	1274C>T	rs3948464	CT	0.30
			CC	0.32
TNF	-308C>T	rs1800629	AG	0.84
			AA	0.80
	-1031C>T	rs1799964	CT	0.85
			TT	**0.13**
	-238A>G	rs361525	AG	**0.001**
			GG	**0.001**
	-863A>C	rs1800630	AC	0.82
			CC	0.23
	-857C>T	rs1799724	TC	0.76

			TT	**0.05**
TNFRSF1A	303A>G	rs4149622	AG	0.71
			GG	0.73
IFNG	874A>T	rs2430561	TA	**0.18**
			TT	**0.13**
IFNGR1	-611C>T	rs1327474	GA	0.85
			AA	0.60
	-56T>C	rs2234711	CT	0.80
			CC	**0.02**
VDR	FokI	rs10735810	CT	0.92
			CC	0.34
	TaqI	rs731236	TC	0.46
			CC	0.86
	BsmI	rs1544410	AG	0.58
			AA	0.30
PTPN22	R630W	rs2476601	GA	0.23
P2X7	1513	rs3751143	TG	0.54
			GG	0.23

*Age is included as a covariate in Poisson regression
